# Perspectives for improvement of *Mycoplasma hyopneumoniae* vaccines in pigs

**DOI:** 10.1186/s13567-021-00941-x

**Published:** 2021-05-08

**Authors:** Dominiek Maes, Filip Boyen, Bert Devriendt, Peter Kuhnert, Artur Summerfield, Freddy Haesebrouck

**Affiliations:** 1 Faculty of Veterinary Medicine, Ghent University, Merelbeke, Belgium; 2 Institute of Veterinary Bacteriology, Vetsuisse Faculty, University of Bern, Bern, Switzerland; 3 Institute of Virology and Immunology, Sensemattstrasse 293, Mittelhäusern, Switzerland; 4 Department of Infectious Diseases and Pathobiology, Vetsuisse Faculty, University of Bern, Bern, Switzerland

**Keywords:** *Mycoplasma hyopneumoniae*, Vaccination, Immune responses, Experimental vaccines, Pig

## Abstract

*Mycoplasma hyopneumoniae* (*M. hyopneumoniae*) is one of the primary agents involved in the porcine respiratory disease complex, economically one of the most important diseases in pigs worldwide. The pathogen adheres to the ciliated epithelium of the trachea, bronchi, and bronchioles, causes damage to the mucosal clearance system, modulates the immune system and renders the animal more susceptible to other respiratory infections. The pathogenesis is very complex and not yet fully understood. Cell-mediated and likely also mucosal humoral responses are considered important for protection, although infected animals are not able to rapidly clear the pathogen from the respiratory tract. Vaccination is frequently practiced worldwide to control *M. hyopneumoniae* infections and the associated performance losses, animal welfare issues, and treatment costs. Commercial vaccines are mostly bacterins that are administered intramuscularly. However, the commercial vaccines provide only partial protection, they do not prevent infection and have a limited effect on transmission. Therefore, there is a need for novel vaccines that confer a better protection. The present paper gives a short overview of the pathogenesis and immune responses following *M. hyopneumoniae* infection, outlines the major limitations of the commercial vaccines and reviews the different experimental *M. hyopneumoniae* vaccines that have been developed and tested in mice and pigs. Most experimental subunit, DNA and vector vaccines are based on the P97 adhesin or other factors that are important for pathogen survival and pathogenesis. Other studies focused on bacterins combined with novel adjuvants. Very few efforts have been directed towards the development of attenuated vaccines, although such vaccines may have great potential. As cell-mediated and likely also humoral mucosal responses are important for protection, new vaccines should aim to target these arms of the immune response. The selection of proper antigens, administration route and type of adjuvant and carrier molecule is essential for success. Also practical aspects, such as cost of the vaccine, ease of production, transport and administration, and possible combination with vaccines against other porcine pathogens, are important. Possible avenues for further research to develop better vaccines and to achieve a more sustainable control of *M. hyopneumoniae* infections are discussed.

## Introduction

*Mycoplasma hyopneumoniae* (*M. hyopneumoniae*) is the most important *Mycoplasma* sp. in swine health management. It is the primary pathogen of enzootic pneumonia, a chronic respiratory disease in pigs, and one of the primary agents involved in the porcine respiratory disease complex [1]. Infections with *M. hyopneumoniae* are highly prevalent in almost all swine producing areas, and they cause significant economic losses due to increased medication use and decreased performance of the pigs [2].

Similar to other mycoplasmas, *M. hyopneumoniae* lacks a cell wall. The organism is very difficult to isolate because of its slow growth and potential overgrowth with other swine mycoplasmas. The pathogen-host interactions are very complex and not fully characterized. The organism is primarily identified on the mucosal surface of the trachea, bronchi, and bronchioles [3]. It affects the mucosal clearance system by disrupting the cilia on the epithelial surface and, additionally, the organism modulates the immune system of the respiratory tract [4]. Therefore, *M. hyopneumoniae* predisposes animals to concurrent infections with other respiratory pathogens.

Control of *M. hyopneumoniae* infections in pig herds can be accomplished by optimizing management, housing and biosecurity practices [5]. Treatment can be done using medication with antimicrobials active against *M. hyopneumoniae*. Antimicrobial medication can limit the consequences of the disease and decrease the infection load [6], but it does not prevent pigs from becoming infected with *M. hyopneumoniae*. Medication with antimicrobials is also discouraged because of the risk of antimicrobial resistance development [7]. Vaccination against *M. hyopneumoniae* has been shown to be a useful tool to control *M. hyopneumoniae* infections. Different inactivated, whole-cell vaccines are commercially available and vaccination is frequently practiced worldwide. In infected herds, vaccination decreases clinical signs and lung lesions due to *M. hyopneumoniae* infections, performance losses of the animals and antimicrobial use. Although the commercial vaccines are widely used and cost-efficient in many farms, they induce only a partial protection and do not prevent infection.

The present paper discusses the complex interaction of *M. hyopneumoniae* with the host and the limitations of the currently available commercial vaccines. Next, the different experimental vaccines that have been developed and tested in mice and pigs are reviewed. Finally, avenues for further research are provided in order to improve *M. hyopneumoniae* vaccination and achieve a more sustainable control of *M. hyopneumoniae* infections.

## Interactions of *M. hyopneumoniae* with the host

### The organism

*M. hyopneumoniae* is a small (0.2–0.4 µm) and pleomorphic organism as it lacks a shape-defining cell-wall. Studies have shown a high diversity at genomic, antigenic, and proteomic level between different strains [8]. The genomes of the earliest described *M. hyopneumoniae* strains (the pathogenic strains 232 and 7448, and the nonpathogenic strain J) were sequenced in 2004 [9, 10]. Since then, the genomes of several other strains have been sequenced. Currently, 23 entirely sequenced *M. hyopneumoniae* genomes are available, 11 already assembled and annotated, and 12 not fully assembled. In general, the genomes are small in size, namely 0.86–0.96 Mb, and in each of them, there are 528 to 691 protein-encoding genes [11]. Despite the small genome, up to 30% of the gene content is still of unknown function [12]. In addition, 20 to 30% of the *M. hyopneumoniae* genes encode surface proteins, the function of many of them is not yet known [12]. The mean GC content, which influences genome organization and gene expression, is low (28.54%) compared to other bacterial species. The low GC content gives *M. hyopneumoniae* a complex transcriptional organization, unique intrinsic terminator stem-loop formation and individual ribonuclease P (RNase P) structure [13].

The small genome of *M. hyopneumoniae* and the limited number of secreted or surface proteins is particularly interesting for use of recombinant DNA technology. However, *M. hyopneumoniae* uses an unusual genetic code. The amino acid tryptophan is not encoded by TGG, but by TGA, which is a stop codon in most organisms [14]. This difference has hampered the expression of genes of *M. hyopneumoniae* containing TGA codons in *E. coli*, the most attractive system used for production of recombinant proteins. However, mutations replacing TGA codons with TGG have been used successfully to solve this problem [15].

### Adherence

Upon inhalation, *M. hyopneumoniae* adheres to the ciliated epithelial cells of the trachea, bronchi and bronchioles underneath the mucous layer. Adhesion is followed by the induction of ciliostasis, loss of cilia, and eventually epithelial cell death [16].

A repertoire of at least 35 M*. hyopneumoniae* proteins have been associated with cell adhesion, including several related to the P97/P102 paralog families and other surface proteins that moonlight as adhesins [17, 18]. The number of *M. hyopneumoniae* adhesins can even be higher, considering that its surfaceome includes more than 290 proteins and that many uncharacterized surface displayed proteins may bear adhesion properties. Different adhesins may vary in abundance at the cell surface between strains. This may be due to differential transcriptional rates of the respective genes, differential translational rates of the corresponding mRNAs and/or post-translational events, including their export to the cell membrane and proteolytic processing. Proteolytic processing of adhesins can shape the bacterial surface architecture [19], generating several adhesin proteoforms, that may exert alternative functions. Some of them are displayed at the cell surface, while others may stay in the cytoplasm or be released from the cell membrane to the extracellular milieu [11].

P97 is one of the most important adhesins of *M. hyopneumoniae* and also the most intensively studied one, and therefore, it has been tested in many experimental vaccines. P97 contains two repeat regions (R1 and R2), located in the C-terminal portion. The sites that are involved in cilium binding are located in the R1 region and at least seven AAKPV/E repeats are required for functional binding. Both R1 and R2 are involved in the attachment of *M. hyopneumoniae* to the extracellular matrix of the respiratory tract [20].

Apart from the cilia-exposed glycans, some swine extracellular matrix molecules, such as fibronectin and plasminogen, also provide binding sites for surface adhesins of *M. hyopneumoniae* [11]. The fibronectin- and plasminogen-binding ability of *M. hyopneumoniae* may mediate subsequent adherence to swine respiratory cilia. Further research is needed to investigate whether adherence to fibronectin and plasminogen may facilitate internalization of *M. hyopneumoniae* and facilitate its traffic via the circulatory system and penetration into host organs, such as liver, kidneys and spleen [21]. The exact role of plasmin in the chronic inflammatory response as observed during *M. hyopneumoniae* infection is unclear, but it may influence the migration of inflammatory cells and stimulate the release of pro-inflammatory cytokines [22].

Extracellular actin is also used as a surface receptor by different proteoforms of *M. hyopneumoniae* P97 adhesin and other proteins, including lipoproteins, glycolytic enzymes, chaperones and translation factors [23]. Apart from extracellular actin, surface proteins of *M. hyopneumoniae* also interact with other cytoskeletal proteins, such as vimentin, keratin, tubulin, myosin, and tropomyosin [24].

### Virulence factors

Adhesion serves as the starting point of infection which is then assisted by other virulence factors. Classical virulence factors like toxins are generally lacking in *Mycoplasma* species. *Mycoplasma hyopneumoniae* can produce H_2_O_2_ in the presence of glycerol in vitro. However, this is strain dependent and the attenuated type strain J does not produce detectable amounts of H_2_O_2_ [25]. Whether production of hydrogen peroxide should be considered as a possible in vivo virulence mechanism in *M. hyopneumoniae* remains to be investigated. Ferrarini et al. [25] showed that *M. hyopneumoniae* is able to take up myo-inositol and use it as an alternative energy source in the absence of glucose. Since myo-inositol is freely available in the serum of pigs, it might be a suitable alternative energy source for *M. hyopneumoniae* residing in the highly vascularized lungs.

Lipid associated membrane proteins (LAMP) have also been implicated in the pathogenicity of mycoplasmas. They interact with the host immune system mainly through Toll-like receptors (TLRs), such as TLR2 [26]. In *M. hyopneumoniae*, whole membrane lipoprotein fractions induced apoptosis in various cell types, including porcine peripheral blood mononuclear cells (PBMCs) [27]. Furthermore, LAMPs activate production of nitric oxide and reactive oxygen species in the host cell [28].

Mycoplasmas need to scavenge nutrients including nucleotides from their environment and therefore, they are well known for their potent membrane nucleases [14]. A well-recognized membrane nuclease is MnuA. Macrophages and neutrophils may form extracellular traps (METs or NETs, respectively), consisting of an interlacement of chromatin fibres rich in DNA, host defense proteins and enzymes, allowing immobilization and killing of invading microbes. In *Mycoplasma bovis (M. bovis)*, MnuA was shown to degrade DNA-based neutrophil and macrophage extracellular traps (NETs and METs, respectively), thereby enabling *M. bovis* to escape these traps [29] and not being killed by these innate immune cells. The nuclease-encoding *mnuA* gene is also present in *M. hyopneumoniae* [30]. Therefore, MnuA could be a surface nuclease that is responsible for the degradation of NETs/METs, allowing *M. hyopneumoniae* to escape the host immune defense and using at the same time the nucleotides and protein synthesis materials as nutrients for proliferation [31].

*Mycoplasma hyopneumoniae* may release extracellular DNA that allows the organism to form biofilms on abiotic and host surfaces [32]. Biofilm formation makes the pathogen more resistant to antimicrobials and the host immune responses. The molecular interactions and cellular processes underlying *M. hyopneumoniae* biofilm formation are thus far mostly unknown.

In *Mycoplasma genitalium*, an immunoglobulin (Ig) G binding protein, called protein M, has been identified. This protein not only fixes IgG very efficiently but also prevents subsequent antigen–antibody binding and subsequent signaling pathways of the bound antibodies [33]. In vitro experiments have shown that genes from *Mycoplasma mycoides* subspecies *capri* encode a *Mycoplasma* Ig binding protein (MIB) and a *Mycoplasma* Ig protease (MIP). The complex of MIB and Ig is necessary for the proteolytic activity of MIP. The two proteins are encoded by two genes and are often detected in multiple copies in various *Mycoplasma* sp., including *M. hyopneumoniae* [34]. Further studies are needed to investigate the role of the MIB-MIP system in virulence and immune evasion of *M. hyopneumoniae*.

Integrative and conjugative elements (ICE) are self-transmissible mobile genetic elements involved in horizontal gene transfer, thereby providing new virulence and/or antibiotic resistance traits. Such ICE have been identified in *M. hyopneumoniae* [35], but their role in encoding virulence traits of *M. hyopneumoniae* remains unclear.

Several large-scale comparisons on the genome, transcriptome, proteome, metabolome and secretome level have been performed in order to investigate virulence and pathogenesis of *M. hyopneumoniae* [24, 36]. Liu et al. reported that besides the known virulence-associated proteins (mainly adhesins), mutations were also found in genes involved in metabolism and growth [36]. *M. hyopneumoniae* is a genome-reduced organism that is characterized by a limited set of biosynthetic pathways, as such it is not surprising that further loss of enzymatic functions might have a large influence on survival and growth of the microorganism. This also holds true for lipoproteins involved in nutrient acquisition [37]. A comprehensive proteome profiling of two *M. hyopneumoniae* strains and *M. flocculare* provided tens of novel candidates to enzootic pneumonia determinants or virulence factors [24].

### Immune responses

The interaction of the pathogen with the immune system of the host is not yet fully elucidated, and it is clear that some components of the immune system may both help and hinder the development of *Mycoplasma*-induced pneumonia [17, 38]. Infection induces the production of pro-inflammatory (e.g. interleukin (IL)-1β, IL-6, TNF-α) and immunoregulatory (e.g. IL-10) cytokines by macrophages, neutrophils and lymphocytes in the lung. This excessive inflammatory response is associated with lymphoid hyperplasia and is considered to be a major driver of lung lesions [39].

#### Innate immune responses

It has been shown that Toll-like receptor 2 (TLR2) and TLR6 are important in the recognition of *M. hyopneumoniae* by porcine alveolar macrophages [40]. The activation of this signal pathway leads to the production of pro-inflammatory cytokines like TNF-α, IL-1β, and IL-6 by alveolar macrophages. Blocking TLR2 and TLR6 receptors led to less TNF-α production by macrophages [41], indicating that alveolar macrophages are involved in inflammatory and innate immune responses during *M. hyopneumoniae* infection. *M. hyopneumoniae* was also demonstrated to strongly activate monocytes and B cells in vitro, with the B cell-activation resulting in a potent polyreactive antibody response [42]. Nevertheless, the role of these responses in protection against *M. hyopneumoniae* infection is still unknown. Also, it is not clear why there is a low neutrophil infiltration upon *M. hyopneumoniae* infection.

Recently, Mucha et al. [43] investigated changes in gene expression of swine epithelial cells of the trachea upon infection with *M. hyopneumoniae*. Among the up-regulated genes, they found several genes related to immune response and inflammation, such as C3 complement, *SAA3*, chemokines (*CXCL2* and *CCL20*) and galectins. These chemokines may attract myeloid cells. The study also suggested that ciliostasis caused by this pathogen might partially be explained by the down-regulation of ciliary genes. The innate immune responses against *M. hyopneumoniae* and mycoplasma in general have been reviewed in more detail elsewhere [4].

#### Humoral responses

After experimental infection, *M. hyopneumoniae*-specific serum IgG antibodies are detected 3–4 weeks post-infection (pi), peak after 11–12 weeks and then decrease very gradually [44]. After booster infection, serum antibody titers clearly increase and then slowly decline again [44]. Interestingly, pigs infected with a highly virulent strain appear to seroconvert earlier than pigs infected with a low virulent strain [45]*. Mycoplasma hyopneumoniae*-specific IgM in serum can be detected as early as 9 days pi under experimental conditions. The percentage of IgM positive pigs peaks at 14 days pi and rapidly decreases afterwards [46]. When infection occurs naturally, seroconversion is usually slower. Local *M. hyopneumoniae*-specific antibodies precede specific serum antibodies following infection but decline faster [47]. *Mycoplasma hyopneumoniae*-specific IgA can be detected in nasal swabs as early as 6 days pi, peak 12–16 days pi and decline steadily afterwards to reach pre-immune levels by day 84 pi.

*Mycoplasma hyopneumoniae*-specific IgG levels in serum induced by vaccination are not correlated with the severity of lung lesions in *M. hyopneumoniae-*infected pigs, suggesting that systemic antibodies play a minor role in protective immunity [48]. The role of mucosal antibodies in the protection against *M. hyopneumoniae* is still unclear. Some studies demonstrated that specific antibody levels in the respiratory tract did not correlate with protection [48, 49], whereas other studies emphasized the role of *M. hyopneumoniae*-specific secretory IgA in preventing adhesion of the microorganism to the ciliated cells of the respiratory tract [50–52]. Furthermore, specific IgG diffusing from the blood into the lung tissue or produced locally in the BALT could opsonize *M. hyopneumoniae*, resulting in phagocytosis by macrophages and neutrophils [53]. However, Deeney et al. [54] recently reported that addition of convalescent porcine sera did not enhance engulfment of *M. hyopneumoniae* by alveolar macrophages in vitro.

#### Cell-mediated responses

T cell-mediated immune responses are generally considered important for protection against Mycoplasmas causing local respiratory infections such as *M. hyopneumoniae* [4]. T cells are key in the regulation of immune responses and have a critical impact on the development of *Mycoplasma*-induced pneumonia [38]. *Mycoplasma pulmonis* challenge studies using various T cell subset-depleted mice indicate that T helper 1 (Th1), Th17 and CD8^+^ T cell responses are responsible for protection against *Mycoplasma* disease. In contrast, Th2 responses are less efficient in controlling the infection and thus contribute to immunopathology [38]. In a *M. hyopneumoniae* vaccination-challenge study resulting in a significant reduction of lung lesions in the vaccinated group, Thacker et al. [50] observed a higher level of IFN-γ-secreting blood lymphocytes in vaccinated pigs compared to non-vaccinated ones before and after experimental infection. In *M. hyopneumoniae*-vaccination studies using a *M. hyopneumoniae*-resistant and a non-resistant pig line, higher serum levels of IFN-γ and IL-17A, but lower levels of IL-4 and CD4^+^ T cells were detected in the resistant line compared to the non-resistant line after vaccination [55]. As IFN-γ, IL-4 and IL-17A are the effector cytokines produced by Th1, Th2 and Th17 cells, respectively [4], these results support the findings obtained in mouse models that Th1 and Th17 responses are responsible for protection against *Mycoplasma* disease. Next to that, Marchioro et al. [53] found a lower CD4^+^/CD8^+^ ratio, and thus a higher relative number of CD8^+^ cells in pigs vaccinated with a commercial *M. hyopneumoniae* bacterin compared to control pigs receiving a physiological saline solution. This supports the hypothesis that CD8^+^ T cells have a protective role in *Mycoplasma* infections and could partially explain the beneficial effects observed after vaccination against *M. hyopneumoniae*.

T helper 1 responses may contribute to protection against *Mycoplasma* infections by IFN-γ-mediated activation of macrophage killing. It is now well-established that Th17 immune responses are important to protect mucosal surfaces, to promote epithelial cell regeneration, mucous and antimicrobial protein production, and the release of neutrophil recruitment [56]. Following a mycoplasma infection, Th17 cells could protect the lung mucosa by attracting other immune cells for pathogen clearance [57] and by elevating secretory IgA levels in the airway lumen [58]. The major characteristic of CD8^+^ cells is killing infected cells [59]. Since there is some evidence that *M. hyopneumoniae* is able to invade porcine epithelial cells [32], this characteristic of CD8^+^ cells might be relevant in the immune response against *M. hyopneumoniae*. Interestingly, studies performed in the *M. pulmonis* mouse model suggest that CD8^+^ T cells might dampen the pro-inflammatory Th cell responses responsible for lung damage and clinical disease [38]. It is suggested that IFN-γ-producing CD8^+^ T cells may skew the responses towards a protective Th1 response. Another possibility could be that CD8^+^ T cells kill antigen presenting cells (APCs), thus reducing the possibility of Th cell activation [38].

## Commercial vaccines against *M. hyopneumoniae*

### Type of vaccines

Commercial vaccines mostly consist of adjuvanted inactivated, whole-cell preparations (for an overview see Maes et al. [60]). Most vaccines are based on the strain J, possibly because it is the type strain of *M. hyopneumoniae* and grows easier in culture medium than recent field isolates. This strain was isolated in 1963 from a field outbreak of enzootic pneumonia in the UK. Commercial bacterin vaccines are licensed either for single or double vaccination, and combinations with porcine circovirus type 2 (PCV-2) or *Glaesserella parasuis* (formerly *Haemophilus parasuis*) are available. Most bacterin vaccines should be administered intramuscularly, but some bacterins are also licensed for intradermal administration. An inactivated vaccine based on soluble antigens of *M. hyopneumoniae* is also commercially available [61].

Attenuated vaccines against *M. hyopneumoniae* have been licensed in Mexico and in China [62]. The vaccine in Mexico is a thermosensitive mutant of *M. hyopneumoniae* (strain LKR) that should be applied intranasally [60]. The attenuated Chinese vaccine strain is derived from a virulent parent strain 168 isolated in 1974 from an Er-hua-nian pig with enzootic pneumonia [63]. This field strain was gradually attenuated by continuous alternating passage through modified Friis medium and pigs. The attenuated strain contains 60 insertions and 43 deletions compared to the original wild type strain. Mutations in genes related to metabolism and growth may contribute to the attenuated virulence, in addition to variations previously described in *M. hyopneumoniae* adhesins (P97, P102, P146, P159, P216, and LppT), cell envelope proteins (P95), cell surface antigens (P36), and secreted proteins and chaperone protein (DnaK) [36]. The Chinese vaccine strain is mostly used by intrapulmonary administration [63]. Residual virulence and/or reversion to increased virulence might represent a risk in case of attenuated vaccines, although the Chinese vaccine has been used already for many years without reported side effects [60].

### Mechanisms of protection upon vaccination

Commercial vaccines induce partial protection against *M. hyopneumoniae* infections. However, the immune mechanisms resulting in partial protection are not fully elucidated. Several studies observed lower levels of the pro-inflammatory cytokines associated with lymphoid hyperplasia and pneumonia lesions, such as TNF-α, Il-6 and IL-1β in *M. hyopneumoniae*-vaccinated pigs compared to non-vaccinated ones [53, 64]. Moreover, vaccinated pigs had a higher number of IL-10-producing cells in their bronchial lymph nodes, which may have an anti-inflammatory effect [53]. Indeed, Vranckx et al. [65] demonstrated that vaccination reduces macrophage infiltration in the BALT of experimentally infected pigs. These findings suggest that vaccination modulates the infiltration of immune cells, as well as the secretion of pro- and anti-inflammatory cytokines, resulting in a reduction of lung lesions. Alternatively, it might also be possible that the reduced inflammatory responses is a consequence of a lower bacterial load [52].

The number of animals seroconverting after vaccination, as well as the antibody levels induced in serum and respiratory tract washings may vary depending on the vaccine composition, administration route, vaccination strategy (single or double vaccination) and the infection status of the animal [60]. The serological response upon a single vaccination is generally lower than after double vaccination. Serum antibodies are usually detected from two to 4 weeks after two-dose vaccination and they remain detectable for weeks to months. In the absence of natural infections that boost the immune system, antibody titers decrease below detection limits 1 to 3 months after vaccination [66]. Early studies indicate no correlation between vaccine-induced serum antibody levels and protection [67], but understanding the role of antibodies requires future investigations. Live-attenuated vaccines applied via the mucosal route could theoretically induce a local IgA response that could prevent colonization but to our knowledge, such data has not been reported.

Several studies found an increase in *M. hyopneumoniae*-specific IFN-γ-secreting cells in the blood and lung tissue of vaccinated animals [53, 64, 68]. These cells, characteristic for local and systemic Th1 responses, are considered to play an important role in vaccine-induced protection.

### Efficacy of commercial vaccines

The major advantages of piglet vaccination relate to increased animal welfare and a decrease of the performance losses due to *M. hyopneumoniae* infections: improvement of daily weight gain (2–8%), feed conversion ratio (2–5%) and sometimes mortality rate. Additionally, shorter time to reach slaughter weight, less variation in slaughter weight (more homogeneous carcasses), reduced clinical signs (coughing), lower prevalence and severity of lesions typically caused by *M. hyopneumoniae* and lower treatment costs, are observed [69]. The currently used vaccines reduce the number of *M. hyopneumoniae* organisms in the respiratory tract [52, 65, 70] and decrease the infection level in a herd [71].

Different factors that may influence vaccine efficacy have been described by Maes et al. [60]. The most important factors include non-compliance with the basic principles of good vaccination practices, stress at vaccination, infections with other pathogens at the moment of vaccination, important co-infections involved in porcine respiratory disease complex, diversity of *M. hyopneumoniae* strains, and maternal immunity. A recent study [72] also hinted at a role of pre-vaccination gut microbiota composition in influencing *M. hyopneumoniae* vaccine responses, although bacterial diversity indexes alone were not predictive for antibody responses among individual pigs.

Drawbacks of the current vaccines are that the protection against clinical signs and lesions typically caused by *M. hyopneumoniae* is often incomplete and vaccination does not prevent colonization. Transmission models under experimental [70] and field conditions [45, 73] also showed that vaccination conferred only a limited and non-significant reduction of the transmission rate of *M. hyopneumoniae*. New vaccines and/or administration routes are therefore needed. A recent pilot study could not demonstrate transmission of *M. hyopneumoniae* between seeder and contact animals in case both had been vaccinated multiple times against *M. hyopneumoniae* [74], suggesting that also the effect of vaccination frequency should be further explored.

## Experimental vaccines

Research on the development of novel vaccines that may offer better protection against *M. hyopneumoniae* infections is ongoing. An overview of the peer-reviewed studies (since 1995) that have investigated different experimental vaccines in mice is shown in Table 1 (vector vaccines), Table 2 (subunit vaccines) and Table 3 (DNA vaccines), while studies that were conducted in pigs are shown in Table 4 (vector vaccines), Table 5 (subunit, membrane proteins, culture supernate vaccines) and Table 6 (bacterin vaccines). Peer-reviewed studies about nucleic acid-based vaccines against *M. hyopneumoniae* in pigs were not found. No publications in peer-reviewed scientific journals have been found about experimental vaccines based on attenuated strains.

**Table 1:** **Experimental vector vaccines against** ***Mycoplasma hyopneumoniae* tested in mice.**

Antigen	Vector	Route	Nb of vaccinations	Humoral response		CMI response^a^	Other/comments	References
				Serum	BALF^b^			
NrdF (R2)	*Salmonella* Typhimurium aroA SL3261	Oral	3	No IgG, no IgA	IgA, no IgG			[75]
P97 (R1)	*Salmonella* Typhimurium aroA CS332 (pro- and eukaryotic plasmid)	Oral	2	No	No	IFN-γ		[76]
NrdF (R2)	*Salmonella* Typhimurium aroA CS332(pro- and eukaryotic plasmid)	oral	2	No	No	IFN-γ response (only with eukaryotic vector)		[77]
P97 (R1)	Adenovirus	IM or IN	2	IM / IN: IgG IM: IgG2a/IgG1 = 4 IN: IgG2a/IgG1 = 1	IM /IN: IgG, IgG1, IgG2a IN: IgA		Serum and BAL inhibited growth of *M. hyopneumoniae*	[78]
P36	*Actinobacillus pleuropneumoniae* SLW36	IM	2	IgG				[79]
P97c	Adenovirus	IM	2	IgG, IgG1, IgG2a, IgG2b, IgG3 IgG2a/IgG1 ≈ 1			P97c may act as immunopotentiator	[80]
P97R1, P46	*Bacillus subtilis*	IN	2	IgG	IgA	IFN-γ, Il-4		[81]

IM, Intramuscular; IN, Intranasal.

^a^CMI cell-mediated immune responses were tested by stimulation of splenocytes.

^b^BALF bronchoalveolar lavage fluid.

**Table 2:** **Experimental subunit vaccines against** ***Mycoplasma hyopneumoniae* tested in mice.**

Antigen	Vaccine type	Adjuvant	Route	Nb of vaccinations	Humoral response		CMI response^a^	Other/comments	References
					Serum	BALF^b^			
P71	Subunit	*Mycobacterium tuberculosis* ESAT-6 recombinant protein	IM	2	IgG1, IgG2a IgG2a/IgG1 ≈1 (with) < 1 (without adjuvant)		IFN-γ, low Il-10		[82]
P97 (R1)	Subunit and its chimeric from with LTB^b^		IM or IN	3	IgG, IgG1, IgG2a (only with chimeric form) IM: IgG2a/IgG1 = 1.2 IN: IgG2a/IgG1 = 0.6	IgA (only with chimeric form)	IFN-γ response (only with chimeric form and IN application)	No anti-R1 antibodies with commercial bacterin	[83]
P97 (R1,R1R2)	Subunit and their chimeric forms with LTB	Montanide IMS 1113	IM	3	IgG	IgA, IgG	IFN-γ	Highest humoral response with chimeric forms	[84]
HSP70	Subunit	Nanoparticles SBa-15 and SBa-16, Aluminium	IP	3	IgG		Only in SBa-15 group; IFN-γ, Il-4, Il-10	Effect SBa-15 comparable to Aluminium	[85]
P97R1, P46, P95, P42	Chimeric – recombinant *E. coli* bacterin	Oil adjuvant (AddaVax™) (for chimeric)	IM	2	IgG, IgG1, IgG2a (IgG2a /IgG1≈0.8)			Antibodies against each antigen; higher response in chimeric group	[86]

IM, Intramuscular; IN, Intranasal; IP, Intraperitoneal.

^a^CMI responses were tested by stimulation of splenocytes.

^b^BALF bronchoalveolar lavage fluid.

^c^LTB B subunit of heat-labile enterotoxin of *E. coli.*

**Table 3:** **Experimental DNA vaccines against *Mycoplasma hyopneumoniae* tested in mice.**

Antigen	Vaccine type	Vector/adjuvant	Route	Nb of vaccinations	Humoral response	CMI response^a^	Other/comments	References
					Serum			
P42	DNA	pcDNA3	IM	2	IgG, IgG1, IgG2a, IgG2b, IgG3 IgG2a/IgG1 = 1.1)	IFN-γ, Il-2, Il-4	Serum inhibited growth of *M. hyopneumoniae*	[87]
P71	DNA	*Mycobacterium tuberculosis* ESAT-6 gene sequences	IM	2	IgG1, IgG2a (higher responses and IgG2a/IgG1 ratio in group with adjuvant)	IFN-γ (higher in group with adjuvant), no Il-10		[88]
P36, P46, NrdF, and P97or P97R1	Subunit (cocktail), DNA, combination	Subunit: Aluminium pcDNA3	Subunit: SC DNA: IM	1	Subunit, combination: IgG against each antigen DNA: IgG only against P46	IFN-γ	Commercial vaccine: no anti-P97 antibodies	[89]
P37, P42, P46, P95	Subunit (cocktail) and DNA	Subunit: Aluminium pcDNA3	IM	2		IFN-γ, lower TNFα and Il-1	Strongest response for P42 and P95 (subunit) and for P46 (DNA)	[90]
P46, HSP70, MnuA antigens	Subunit (cocktail) and DNA	Subunit: Freund’s adjuvant pcDNA3.1	Subunits: IP DNA: IM	3	IgG	IFN-γ, Il-10, no Il-4	Mixed response, but predominantly Th1	[91]

IM, Intramuscular; SC, Subcutaneous; IP, intraperitoneal.

^a^CMI responses were tested by stimulation of splenocytes.

**Table 4:** **Efficacy and immune responses of experimental vector vaccines against *Mycoplasma hyopneumoniae* tested in pigs.**

Antigen	Vector	Route	Number of vaccinations	Significant decrease of			Humoral response		CMI response^a^	Other/comments	References
				Lung lesions	Clinical signs	*M. hyopneumoniae* numbers	Serum	BALF^b^			
NrdF (R2)	*S.* Typhimurium aroA SL3261^c^	Oral	2	Yes			No	IgA	Yes	Higher ADG^b^; IgA only after challenge	[94]
P97 (R1R2)	*E. rhusiopathiae* strain YS-19^c^	Intranasal	2	Yes		Yes	No		Yes		[95]
P97 (R1R2)	*E. rhusiopathiae* strain Koganei 65–0.15	Oral	3	Yes		No	IgG	IgA	Yes	IgG and IgA only after challenge	[96]
P97 (R1R2)	Adenovirus	Intranasal	2	Yes		Yes	IgG, IgA	IgA, IgG (saliva)	Yes	higher ADG; serum inhibited *M. hyopneumoniae* growth; minor decrease of lung inflammation Commercial vaccine: no anti-P97 antibodies, better protection	[97]

^a^CMI responses were tested by stimulation of peripheral blood mononuclear cells (PBMCs) and calculating stimulation indexes.

^b^BALF, bronchoalveolar lavage fluid; ADG, average daily gain.

^c^*S.* Typhimurium: *Salmonella* Typhimurium; *E. rhusiopathiae: Erysipelothrix rhusiopathiae.*

**Table 5:** **Efficacy and/or immune responses of experimental vaccines (subunit, membrane proteins, culture supernate) against *Mycoplasma hyopneumoniae*** **tested in pigs.**

Antigen	Vaccine type	Adjuvant/carrier	Route	Nb of vaccinations	Challenge infection	Decrease of			Humoral response		CMI response^b^	Other/comments	References
						Lung lesions	Clinical signs	*M. hyopneumoniae* numbers	Serum	BALF ^c^			
P97	Subunit	Complete Freund’s adjuvant	IM	2	Yes	No	No		Yes^d^				[98]
J strain	Membrane proteins	Five different adjuvants^e^	IM-IP	2	Yes	Yes^e^			IgG, IgA	IgA, IgG		No effect on ADG; Humoral response in BALF: only after challenge, greater in IM groups	[48]
Strain 1986–1	Cell-free culture supernate	Al(OH)3	IM	2	Yes	Yes		Yes	IgG (low)				[99]
Strain 1986–1	Cell-free culture supernate	Al(OH)3	IM	2	Yes	Yes		Yes		IgA, IgG		Less macrophages, lymphocytes and TNFα in lungs Humoral response in BALF only after challenge	[100]
P97 (RR1) ^a^	Chimeric subunit with *Pseudomonas* exotoxin A		SC (mice)-IM (pigs)	2	No				IgG				[101]
P97 (R1R2)	Chimeric subunit with N-terminal region *A. pleuropneumoniae* ApxIII	Freund’s adjuvant (mice), Al(OH)3 (pigs)	SC (mice)-IM (pigs)	2	Yes (pigs)	Yes	Yes		IgG1, IgG2a (mice)		Higher IFN-γ and IL-4 (mice)	Also protection against *A. pleuropneumoniae*	[102]
P97, P42, NrdF	Chimeric subunit	LTB^f^	IM-IN	2	Yes	No	No	No	IgG	IgA		IgA in BALF only after challenge Commercial vaccine: lower serological response, better protection	[21]
P102 and 8 fragments of P97 /P102 paralogs	Subunit	Al(OH)3 and polymer based (Montanide™)	IM	3	Yes	No	No	No	IgG	No IgA		Less cilia damage, less Il-1, Il-6, TNFα in BALF Commercial vaccine: lower serological response, better protection	[52]

IM, Intramuscular; SC, Subcutaneous; IN, Intranasal; IP, intraperitoneal.

^a^Study was done in pigs and mice.

^b^CMI response was tested by stimulation of splenocytes.

^c^BALF bronchoalveolar lavage fluid.

^d^tested by Western blotting for reactivity with whole cell lysates.

^e^The membrane preparations were formulated with one of the following adjuvants: 1) Auspharm oil, 2) Alhydrogel, 3) Algammulin, 4) DEAE dextran with Auspharm oil, 5) DEAE dextran with mineral oil. Lung lesions were significantly reduced with all formulations compared to non-vaccinated pigs, but there were no significant differences between the formulations.

^f^LTB B subunit of heat-labile enterotoxin of *E. coli.*

**Table 6:** **Efficacy and/or immune responses of experimental bacterin vaccines against** ***Mycoplasma hyopneumoniae*** **tested in pigs.**

Mycoplasma strain	Adjuvant/carrier	Route	Nb of vaccinations	Challenge infection	Decrease of			Humoral response		CMI response^a^	Other/comments	References
					Lung lesions	Clinical signs	*M. hyopneumoniae* numbers	Serum	BALF^b^			
PRIT-5	Micro-encapsulated (oral); Al(OH)3 (IM)	Oral – IM	3	Yes	Yes			IgG, IgA	IgA (nose, saliva)		Highest humoral responses in oral group	[103]
PRIT-5	Micro-encapsulated (oral); Al(OH)3 (IM)	Oral – IM – IM/oral	3	Yes	Yes			IgG, IgA	IgA (nose, saliva)		Highest humoral responses in oral/combined groups Best protection in IM/oral group	[104]
F7.2C ^c^	Lipo_AMP	IM-IM	2	No				IgG, no IgA	no IgA	Th1 – no Th17	E: 3/3 – L: 3/3 ^d^	[105]
	Lipo_TLR	IM-IM								no Th1 – no Th17	E: 3/3 – L: 3/3	
	Lipo_DDA:TDB	ID-IM								Th1 – no Th17	E: 2/3 – L: 1/3	
	SWE_TLR	IM-IM								Th1 – no Th17	E: 3/3 – L: 1/3	
	PLGA_TLR	IM-IM						No IgG/IgA	no IgA	no Th1 – Th17	E: 0/3 – L: 0/3	
F7.2C	Lipo_DDA:TDB	IM-IM	2	Yes	Yes	Yes	Yes	IgG, IgA	IgA	Th1, Th17, CD8 +	Also reduction of microscopic lung lesions IgA response only after challenge Highest efficacy in SWE_TLR group	[106]
	SWE_TLR									Th1, Th17, CD8 +		
	PLGA_TLR									Th1, no Th17, CD8 +		

IM, Intramuscularly; ID, intradermally.

^a^CMI responses were tested by stimulation of peripheral blood mononuclear cells (PBMCs).

^b^BALF bronchoalveolar lavage fluid.

^c^The bacterin was formulated with 1) cationic liposomes + STING ligand c-di-AMP (Lipo_AMP), 2) cationic liposomes with TLR ligands targeting TLR1/2, TLR7/8 and TLR9 (Lipo_TLR), 3) cationic liposome formulation with the MINCLE agonist trehalose 6,6-dibehenate DDA:TDB liposomes (Lipo_DDA:TDB), 4) squalene-in-water emulsion with the same TLR ligands (SWE_TLR), 5) microparticle formulation with the same TLR ligands (PLGA_TLR).

^d^Number of induced blood transcriptional modules (BTM) by the vaccine group. In total, three early (E) (day 0 to 1) and three late (L) (day 1 to 7) different BTM) were measured: early inflammatory, early IFN type I, early myeloid cell/DC, late cell cycle, late T/NK-cell, late Ig.

### Experimental vaccines studied in mice

The studies in mice focused on the construction and development of the vaccines and the evaluation of the immune responses. As *M. hyopneumoniae* is only causing disease in pigs, the efficacy of the vaccines was not tested in mice. All studies assessed the humoral responses in serum, many also included cell-mediated immunity (CMI), whereas the humoral response in the BAL fluid was less frequently examined.

In terms of antigen selection, most studies included outer membrane proteins that are immunogenic and/or considered important for adhesion such as P97, P46, P71 and P95. However, also other antigens were tested such as NrdF, P36, HSP70, P42, P37 and MnuA. NrdF is an essential enzyme for metabolic processes. It catalyzes the conversion of ribonucleoside diphosphates to deoxyribonucleoside diphosphates, an essential step in DNA replication [75]. P36 or L-lactate dehydrogenase is an early immunogenic protein [79]. HSP70 is a 70 kDA heat shock protein that has been used successfully as a vaccine antigen for other pathogens such as *Salmonella* Typhi in mice and *Mycobacterium avium* subsp. *paratuberculosis* in cattle [85]. P42 is also a heat shock protein and is a member of the HSP70 family [86]. It is highly expressed under stress conditions, and specific antibodies against P42 were able to block the growth of *M. hyopneumoniae*. P37 is a lipoprotein that belongs to the ATP-binding cassette (ABC) transporters [90], whereas MnuA is a membrane nuclease and considered a potential virulence factor [91]. The antigens in the vaccines were produced by recombinant DNA technology using *E. coli* as expression system.

Most vector vaccines were based on bacterial vectors namely *Salmonella*, *Actinobacillus* and *Bacillus*, while two studies used adenovirus vectors. The subunit vaccines were based on single antigens or different antigens that were mixed with an adjuvant. In three of the five studies, the antigens were constructed as a chimeric protein. Subunit vaccines were used in combination with adjuvants based on oil, aluminium hydroxide, the B subunit of heat-labile enterotoxin of *E. coli* (LTB) or *Mycobacterium tuberculosis* ESAT-6 protein. Vaccination with DNA constructs encoding potential antigens might be promising, as in general, DNA constructs are stable, easy to handle and can be administered via various routes.

Most vaccines in mice were applied two or three times, and were administered via the parenteral route, mainly intramuscularly, although oral (vector vaccines), intranasal and intraperitoneal administration routes were evaluated as well.

The major outcome parameters of the studies in mice were humoral responses in serum and in some studies also in BAL fluid, and CMI responses. Humoral responses in serum were assessed by measuring IgG against the antigens included in the vaccine, some studies also measured isotypes of IgG. In general, IgG1 in mice is indicative of a Th2 response, whereas IgG2a is predominantly produced during a Th1-type response [92]. CMI responses were examined by isolation and subsequent stimulation of splenocytes with the respective antigens. IFN-γ production by splenocytes was measured in every study, other cytokines (IL-10, IL-4, TNF-α) were less frequently assessed.

The immune responses with the orally applied *Salmonella* vector vaccine were poor, IgA was only found in BAL fluid in one study and IFN-γ production in another study (Table 1). The immune responses with the other vector vaccines that were administered intramuscularly or intranasally were more pronounced, but also variable between studies. When used under field conditions, vector vaccines might have the problem that immunity is present in the animals against the vector micro-organism e.g. *Salmonella*, leading to lower expression of and immunity against the carried antigen.

The immune responses following administration of subunit vaccines (Table 2) were more consistently measured compared to those of the vector vaccines. Mostly, mixed Th1/Th2 responses were obtained. Immune responses were generally more pronounced when different antigens were presented in a chimeric form [83, 84, 86]. Possibly, in these studies, the epitopes were better accessible by the immune system in the chimeric proteins. Immunization by the intramuscular route appeared to favor a Th1-type response (higher IgG2/IgG1 ratio), while the intranasal route induced a mixed response [78, 83]. Adding *Mycobacterium tuberculosis* ESAT-6 protein or the gene sequences as adjuvant also shifted the immune response towards a Th1-type response [82].

Vaccination with the DNA constructs elicited mixed responses (Table 3), with predominance for Th1-type responses [87, 91]. IgA in BAL fluid was not tested. Th1-type responses stimulate B-cells to produce strongly opsonizing antibodies, such as IgG2a and IgG2b in mice, enhancing the phagocytosis by alveolar macrophages. In mice, IFN-γ, produced by Th1-type cells, induces nitric oxide release from monocytes/macrophages. In case genes coding for multiple antigens were included [89–91], the responses were variable between the antigens, and could also be different when compared to the response obtained by the respective single subunit vaccines.

Overall, some studies showed promising immune responses in mice. However, many phenotypic as well as functional differences in immune cell populations exist between the porcine and the murine immune system [93]. Therefore, studies in mice should be considered as preliminary and need to be validated in pigs.

### Experimental vaccines studied in pigs

The experimental vaccine studies in pigs are summarized in Tables 4, 5 and 6. Most of the studies in pigs used an experimental challenge infection model, allowing to assess the efficacy of the vaccines. CMI responses in pigs were tested by stimulation of PBMCs and subsequently measuring the stimulation index (vector vaccines) or specific cytokine responses (bacterin vaccines). No CMI responses were measured in the studies with the subunit vaccines.

The antigen selection for the vector and subunit vaccines mostly included the P97, alone or in combination with other antigens e.g. NrdF, P42 or P102. One study used membrane proteins [48] and two studies used cell-free culture supernatant of *M. hyopneumoniae* [99, 100]. The bacterin vaccines were based on the strains PRIT-5 [103, 104] and F7.2C [105, 106]. Subunit and bacterin vaccines were mostly used in combination with an adjuvant such as aluminium hydroxide, Freund’s adjuvant (both complete or incomplete), bacterial toxin subunits (LTB), oil or polymer-based adjuvants, macrophage-inducible C-type lectin (MINCLE) agonist, the stimulator of interferon genes (STING) ligand and a combination of different TLR ligands. Vectors used were *Salmonella* Typhimurium, *Erysipelothrix rhusiopathiae* and adenovirus. Vector vaccines were administered orally or intranasally, while most subunit and bacterin vaccines were administered parenterally. In two studies, the bacterin was provided orally in microspheres [103, 104]. All experimental vaccines in pigs were applied two or three times.

In terms of efficacy, 11 out of 14 studies showed a significant reduction of the lung lesions caused by *M. hyopneumoniae* upon challenge infection. Two out of five studies showed a reduction of clinical signs (coughing), and five out of eight a reduction of the *M. hyopneumoniae* load in the BAL fluid. Three studies [21, 52, 97] included a commercial bacterin as control. Their efficacy was each time better than for the experimental vaccines, although the immune responses against the epitopes of the experimental vaccines were lower or absent upon vaccination with the commercial vaccines. This might be due to the fact that some proteins of the bacterins might not be expressed or only expressed in negligible amounts under culture conditions [107], or to the formulation or the adjuvant. Proteomic analysis by Pendarvis et al. [108] showed that only 483 of 691 (70%) proteins in *M. hyopneumoniae* 232 strain were expressed under in vitro culture conditions. This might be one of the reasons of the incomplete protection induced by commercial vaccines. Nonetheless, the efficacy of the experimental vaccines was even lower, suggesting that immunity induced by the antigens selected for preparing these vaccines is not sufficient for improved protection, although it is well known that these antigens like P97 have important roles in the pathogenesis of *M. hyopneumoniae* infections.

All studies could demonstrate specific IgG responses in the serum, except for two vector vaccines [94, 95]. In all studies that had measured mucosal IgA responses in the BAL fluid, IgA could only be detected after challenge infection, but not after vaccination prior to challenge (four studies used intramuscular, two studies oral administration). In three studies [97, 103, 104], IgA was detected in the saliva and/or nose after vaccination and prior to challenge infection. In these studies, pigs were vaccinated intranasally, orally or intramuscularly. In the pigs vaccinated intramuscularly, IgA levels in the saliva and especially in the nose were low [103, 104].

In terms of CMI responses, all studies with the vector vaccines could demonstrate significant responses upon specific stimulation of PBMCs. None of the studies with subunit vaccines (Table 5) describe assessment of CMI responses. For the bacterin vaccines, CMI responses were only assessed in the studies of Matthijs et al. [105, 106]. The study of Matthijs et al. [105] did not apply challenge infection, but made a detailed assessment of the immune responses of five different bacterin formulations. They were all based on a more recently isolated field strain of *M. hyopneumoniae* compared to the type strain J, in combination with different novel adjuvants. These included different Toll-like receptor ligands (targeting TLR1/2, TLR7/8, TLR9), the MINCLE agonist trehalose 6,6-dibehenate (TDB), and STING ligand cyclic diadenylate monophosphate (c-di-AMP). They were used in combination with different carriers namely liposomes, polylactic-co-glycolic acid (PLGA) microparticles or squalene-in-water emulsion (SWE) (see Table 6). The responses were variable depending on the group. Lipo_DDA:TDB, Lipo_AMP and SWE_TLR significantly induced Th1 cytokine-secreting T cells. Only PLGA_TLR appeared to induce Th17 cells, but was unable to induce serum antibodies.

The study of Matthijs et al. [106] also applied a systems vaccinology approach developed for humans and adapted the approach for use in pigs. The transcriptomic analyses demonstrated that the induction of inflammatory and myeloid cell blood transcriptional modules (BTM) in the first 24 h after vaccination correlated well with serum antibodies, while negative correlations with the same modules were found seven days post vaccination. Furthermore, many cell cycle and T cell BTM upregulated at day seven, correlated positively with adaptive immune responses. When comparing the delivery of the identical TLR ligands with the three formulations, SWE_TLR was shown to be more potent in the induction of an early innate immune response, while the liposomal formulation more strongly promoted late cell cycle and T cell BTM. For the PLGA formulation, there were signs of a delayed and weak perturbation of these BTM. The study of Matthijs et al. [105] demonstrated the utility of transcriptome-based systems immunology analyses in identifying early immune signatures in the blood and in unraveling the mechanistic events leading to the stimulation of adaptive immune responses after vaccine injection in pigs.

In a subsequent study, Matthijs et al. [106] assessed the efficacy of three bacterin formulations that were able to induce a Th1 or Th17 response in the previous study, namely a cationic liposome formulation with the Mincle receptor ligand trehalose 6,6-dibehenate (Lipo_DDA:TDB), a squalene-in-water emulsion with Toll-like receptor (TLR) ligands targeting TLR1/2, TLR7/8 and TLR9 (SWE_TLR), and a poly(lactic-co-glycolic acid) microparticle formulation with the same TLR ligands (PLGA_TLR). All three formulations showed promising results, but the highest CMI responses were obtained in the SWE_TLR group. This experimental vaccine also showed the best efficacy in terms of reducing clinical signs, lung lesions and bacterial load in the lung.

Overall, the research with the different experimental vaccines in pigs suggests that the construction, the type of antigens, the adjuvant and/or carrier, and the route and frequency of immunization could induce variable immune responses and efficacy. Based on the available studies, it is not possible to unravel the effect of one of these characteristics e.g. antigen, adjuvant and administration route, as the studies were not designed for this purpose and differ each time in more than one characteristic. In addition, the studies listed in Tables 1 and 2 also point to a large variation in individual animal responses, even if the same vaccine is used under the same conditions.

## Avenues for further research

As far as this was investigated, the experimental vaccines that have been tested in pigs (Tables 4, 5, 6) do not provide superior protection compared to the commercially available bacterin vaccines. Therefore, further research on the development of new experimental vaccines and validation of the efficacy of promising vaccines in pigs is needed.

From an immunological point of view, a major challenge is to induce a protective immunity at the mucosal surface of primary adherence and multiplication of *M. hyopneumoniae*, namely the ciliated epithelium of the trachea, bronchi and bronchioli. From a practical viewpoint, also aspects such as cost of the vaccine, ease of production, transport and administration, differentiation of infected from vaccinated animals (DIVA), and possible combination with other vaccines are important.

### Administration route

Administration of the antigens intranasally or via aerosols might be the most appropriate routes to accomplish mucosal protection. The number of experimental vaccines that have evaluated this route of immunization is still very limited. This is mainly due to important difficulties that are encountered by using the mucosal route such as: (1) the antigen should be present at and below the mucosal barrier in sufficient amounts allowing sufficient capturing by antigen presenting cells, (2) mucosal tolerance mechanisms should be overcome, (3) protective immune mechanisms should be activated and, (4) minimal/or no influence on mucosal functionality should occur. Mucosal adjuvants e.g. microbe-derived substances, including TLR ligands, could be crucial in reaching these goals [109, 110]. Heterologous prime-boost regimes e.g. intramuscular followed by mucosal administration or vice versa, allowing a broader or more variable triggering of the immune system, might also be considered. Vaccination via aerosols is probably technically possible, as nebulization of lung homogenate positive for *M. hyopneumoniae* to gilts under field conditions, resulted in infection of the animals with this pathogen [111]. Also, Feng et al. [62] showed that the attenuated vaccine used in China could be administrated as an aerosol.

Providing the antigen orally might also induce mucosal immunity in the respiratory tract via the common mucosal immune system [112]. In the studies of Lin et al. [103, 104], microencapsulated inactivated whole-cell *M. hyopneumoniae* vaccine was administered orally to pigs via a tube into the esophagus on three occasions. IgA was detected in nasal secretions and saliva, but IgA was not measured in BAL fluid. However, lung lesions following challenge infection were significantly reduced in vaccinated pigs, pointing to protective immunity in these animals. Placing a tube in the esophagus is labor intensive, time consuming and not animal welfare friendly. The stability of the antigen during the mouth-stomach-intestine passage might also be an issue. Therefore, further research should focus on whether *M. hyopneumoniae* vaccines mixed in the diet and consumed by the pigs can also provide protection. Such a method might require a higher antigen dose and also the stability of the antigen in the feed might be an issue. However, it would be animal welfare friendly and require much less time and labor than administration of the vaccine via a tube to each individual animal.

Most experimental vaccines were applied via the parenteral route, mostly intramuscularly. The studies using parenteral administration however could not demonstrate measurable IgA responses in the BAL fluid prior to challenge, and therefore, this route is less suitable for inducing mucosal humoral responses. However, higher *M. hyopneumoniae*-specific IgA levels were observed in respiratory tract washings of parenterally vaccinated pigs compared to non-vaccinated pigs after challenge infection, indicating an anamnestic immune response. This suggests that priming of the mucosal immune system is possible after parenteral vaccine administration. Intradermal vaccination directly targets epidermal Langerhans cells and dermal dendritic cells, which are essential for efficient T and B cell priming. In this sense, intradermal vaccination against *M. hyopneumoniae* can be an asset, as more of these specialized APCs are present in the skin compared to muscle tissue [113]. In addition, no needles are used as the vaccine is administered intradermal via pressure, which may reduce the risk for iatrogenic infections.

### Antigen

Bacterial genes and antigens involved in survival of the bacterium in the host and/or that render the bacterium harmful to the host could be included in vaccines or could be targets for attenuation to develop attenuated vaccines. Many experimental vaccine studies have included P97 and other proteins that are important for adherence or metabolism. However, subunit vaccines based on the most important adhesion namely P97, did not provide sufficient protection. The adhesion process is complex and involves many different adhesins, therefore, including one or only a few antigens might be insufficient. This might also explain why bacterins, which include a broad array of antigens, perform better in terms of efficacy than subunit vaccines. Further research on subunit vaccines should therefore not focus on one or a few antigens, but include different and carefully selected antigens.

### Adjuvant and carrier

Adjuvants are incorporated into vaccines to increase and direct the immunogenic responses to antigens. Many adjuvants activate the innate immune system through pattern-recognition receptors (PRRs) present in immune cells. Receptor-ligand interactions lead to expression of genes that encode cytokines, chemokines, and costimulatory molecules responsible for priming, expansion, and polarization of immune responses [114]. Adjuvants can also induce adaptive immune responses either by enhancing T cell responses, by stimulating humoral immunity or both. As Th1 and Th17 responses are considered important for protection against *M. hyopneumoniae* infection, adjuvants that stimulate this arm of the immune system should preferably be selected [105]. Commonly used adjuvants and their modes of action along with strengths and weaknesses have been reviewed by Bastola et al. [114].

Adjuvants not only increase the immunogenic responses, but can also lead to adverse reactions such as inflammation at the injection site, granuloma and sterile abscess formation, malaise, fever and other systemic reactions. Apart from the study of Matthijs et al. [105], information about safety of adjuvants in experimental *M. hyopneumoniae* vaccines is very scarce. Research on adjuvant development should therefore not only identify more inflammatory adjuvants, but also try to separate the potency of adjuvants from the adverse effects they can induce.

### Attenuated vaccines

Attenuated vaccines have great potential to better stimulate a mucosal immune response at the respiratory tract compared to parenterally administered vaccines. However, apart from the two current commercially available attenuated *M. hyopneumoniae* vaccines, very few studies have focused on developing experimental attenuated *M. hyopneumoniae* vaccines. This might be due to the difficult isolation and cultivation characteristics of *M. hyopneumoniae*. In addition, reversion of virulence is always a concern in case of attenuated vaccines*.* One study [115] tested whether a low virulent *M. hyopneumoniae* strain could be used as a potential vaccine. However, infection with this strain did not protect piglets against infection with a highly virulent *M. hyopneumoniae* isolate 1 month later, suggesting that low virulent strains might not be suitable as such to be used as vaccines. Current technology allows however to selectively delete specific genes important for replication or virulence in pathogenic organisms, which might be useful for the development of attenuated vaccines against *M. hyopneumoniae*. At this stage however, these genes are not yet fully elucidated, and therefore, further research is needed to investigate genes of *M. hyopneumoniae* involved in pathogenesis and virulence.

## Conclusions

The very complex interaction of *M. hyopneumoniae* with the respiratory tract and the immune system and the fact that naturally elicited immune responses upon infection are not able to rapidly clear the pathogen from the animal, make the search for protective immune responses and efficacious novel vaccines challenging. Different experimental vaccines against *M. hyopneumoniae* have been developed and tested in mice and pigs. Most of them were based on the P97 adhesin or other factors considered important in the pathogenesis, or were bacterins combined with novel adjuvants. As cell-mediated and likely also mucosal humoral responses are important for protection, new vaccines should aim to target these arms of the immune response. Very few research has been directed towards the development of attenuated vaccines, although such vaccines may have great potential.

## Abbreviations

ABC: ATP-binding cassette
APC: Antigen presenting cell
BAL: Bronchoalveolar lavage
BTM: Blood transcriptional modules
c-di-AMP: Cyclic di-adenylate monophosphate
CMI: Cell-mediated immunity
DC: Dendritic cell
DDA:TDB: Dimethyl dioctadecylammonium:trehalose 6,6-dibehenate
DIVA: Differentiation of infected from vaccinated animals
HSP: Heat shock protein
ICE: Integrative and conjugative elements
IFN: Interferon
Ig: Immunoglobulin
IL: Interleukin
IN: Intranasal
LAMP: Lipid associated membrane proteins
LTB: Heat-labile enterotoxin of *E. coli*
*M. hyopneumoniae*: *Mycoplasma hyopneumoniae*
MET: Macrophage extracellular trap
MIB: *Mycoplasma* Ig binding protein
MINCLE: Macrophage-inducible C-type lectin
MIP: *Mycoplasma* Ig protease
NET: Neutrophil extracellular trap
NK: Natural killer
NrdF: Ribonucleoside-diphosphate reductase
PBMC: Peripheral blood mononuclear cell
PGLA: Poly(lactic-*co*-glycolic acid)
STING: Stimulator of interferon genes
SWE: Squalene-in-water
Th: T helper
TLR: Toll-like receptor
TNF: Tumor necrosis factor
